# Changes in peripheral immune cell numbers and functions in octogenarian walkers – an acute exercise study

**DOI:** 10.1186/s12979-017-0087-2

**Published:** 2017-02-22

**Authors:** Kornelis S. M. van der Geest, Qi Wang, Thijs M. H. Eijsvogels, Hans J. P. Koenen, Irma Joosten, Elisabeth Brouwer, Maria T. E. Hopman, Joannes F. M. Jacobs, Annemieke M. H. Boots

**Affiliations:** 1 Departments of Rheumatology and Clinical Immunology and Translational Immunology Groningen (TRIGR), University of Groningen, University Medical Center Groningen, Hanzeplein 1, 9700RB Groningen, The Netherlands; 2 Department of Physiology, Radboud University Medical Centre, Nijmegen, The Netherlands; 3 Department of Laboratory Medicine, Laboratory Medical Immunology, Radboud University Medical Centre, Nijmegen, The Netherlands

**Keywords:** T cells, Recent thymic emigrants, NK cells, Monocytes, Ageing, Immune System

## Abstract

**Background:**

Age-related changes of the immune system, termed immunosenescence, may underlie the increased risk of infections and morbidity in the elderly. Little is known about the effects of acute exercise on peripheral immune parameters in octogenarians. Therefore, we investigated acute exercise-induced changes in phenotype and function of the immune system in octogenarians participating in the 2013 edition of the Nijmegen Four Days Marches. Blood sampling was performed at baseline and immediately after 4 days of the walking exercise (30 km/day). A comprehensive set of adaptive and innate immune traits were enumerated and analyzed by flow-cytometry. Peripheral blood mononuclear cells, isolated before and after walking were stimulated with LPS and supernatants were analysed for IL-1β, IL-6, IL-8 and TNF-α concentrations by ELISA. CMV serostatus was determined by ELISA.

**Results:**

The walking exercise induced a clear leucocytosis with numerical increases of granulocytes, monocytes and lymphocytes. These exercise-induced changes were most profound in CMV seropositive subjects. Within lymphocytes, numerical increases of particularly CD4+ T cells were noted. Further T cell differentiation analysis revealed profound increases of naïve CD4+ T cells, including naïve Treg. Significant increases were also noted for CD4+ memory T cell subsets. In contrast, only slight increases in naïve and memory CD8+ T cell subsets were detected. Exercise did not affect markers of immune exhaustion in memory T cell subsets. NK cells demonstrated a numerical decline and a change in cellular composition with a selective decrease of the mature CD56^dim^ NK cells. The latter was seen in CMV seronegative subjects only. Also, a higher IL-6 and IL-8 production capacity of LPS-stimulated PBMC was seen after walking.

**Conclusion:**

In this exceptional cohort of octogenarian walkers, acute exercise induced changes in immune cell numbers and functions. A clear response of CD4+ T cells, rather than CD8+ T cells or NK cells was noted. Remarkably, the response to exercise within the CD4+ T cell compartment was dominated by naïve CD4+ subsets.

**Electronic supplementary material:**

The online version of this article (doi:10.1186/s12979-017-0087-2) contains supplementary material, which is available to authorized users.

## Background

Age-related changes of the immune system may contribute to increased vulnerability for infectious disease, impaired responses to vaccination and the development of late-onset chronic inflammatory diseases [1–3]. This process, termed immunosenescence, is caused by changes in both the adaptive and innate immune system. The causes underlying immunosenescence may be largely environmental as a recent systems level analysis in healthy twins revealed that non-heritable (environmental) factors rather than heritable factors shape the immune system over time [4]. In particular, the broad impact of human Cytomegalovirus (CMV) infection, a non-heritable factor, on the phenotype of the immune system was demonstrated, thereby confirming previous findings [5, 6]. The effects of exercise as another non-heritable (behavioural) factor on the phenotype of the ageing immune system has been less well studied.

The development of immunosenescence includes the decline of naïve T cells due to thymus involution, increases in late-stage effector memory T cells, decreased CD4/CD8 ratio’s and the development of immune exhaustion [7, 8]. These changes result in inadequate T cell help to B cells, thereby affecting the development of productive immune responses. CMV infection is known to accelerate immune ageing through oligoclonal expansion of CMV- specific CD8 effector memory T cells [5, 6]. In addition, several studies report on increases in T regulatory cells (Treg) leading to increased Treg/Teffector ratio’s in healthy elderly which may further add to the development of immunosenescence [9–11].

Whilst adaptive immune responses decline with age, the activity of the innate immune system appears to increase with age. This is evidenced by numerical increases in natural killer (NK) cells and monocytes and by increased serum levels of acute phase proteins and inflammatory cytokines such as interleukin-1 β (IL-1β), Interleukin-6 (IL-6), interleukin-8 (IL-8) and Tumor Necrosis Factor-α (TNFα) [12, 13]. The molecular mechanisms underlying this chronic, low grade inflammation (coined inflamm-ageing) are currently unknown but may be associated with an altered innate response to an altered gut microbiota [12, 14].

NK cells are key in the protection against infection and cancer. Ageing-associated alterations have shown an increase in the more mature CD56^dim^ subset and a decline of the immature, CD56^bright^ NK subset, irrespective of CMV infection [15]. CD56^dim^ NK cells are the most abundant subset in the blood, and demonstrate a higher cytotoxicity, whereas the CD56^bright^ NK cells demonstrate higher cytokine production. CMV chronic infection is associated with an expansion of a “memory-like” (CD56^dim^) NK cell subset characterized by NKG2C expression and lack of NKG2A [15].

Physical activity and exercise have profound effects on the immune system and contribute to health, well-being and longevity [16, 17]. Single bouts of exercise induce a prominent leukocytosis followed by a redistribution of immune effector cells to the tissue compartments [18]. This biphasic response to exercise may enhance the immune response against pathogens in the lymph nodes and in peripheral tissues (e.g. skin, mucosa, lungs). In adult individuals, exercise-induced lymphocytosis is largely attributed to NK cells and CD8 effector memory T cells [19, 20]. Interestingly, these subsets share functional characteristics such as cytotoxicity and tissue migration, which are important in immunosurveillance. Notably, CMV serostatus was found to influence the magnitude and the kinetics of the NK and CD8+ memory T cell responses to exercise [21, 22]. To our knowledge, the effects of acute exercise on the phenotype and function of the immune system in octogenarians have not yet been documented.

In the current study we thus investigated the effects of acute exercise on a comprehensive set of adaptive and innate immune traits in a small cohort of 20 elderly octogenarian walkers participating in the 2013 Nijmegen Four Days Marches. All participants walked a total 120 km in four consecutive days (4 x 30 km) at a self-selected pace. Blood samples were drawn at baseline and immediately after completion of the march at day 4. A post hoc analysis was performed on the contribution of CMV serostatus to exercise-induced immune changes.

## Methods

### Participants

Twenty elderly male and female participants (mean age 81.3 ± 1.9 years) of the 2013 Nijmegen Four Days Marches volunteered to participate in our study. The Nijmegen Four Days Marches represents the largest mass participation walking event in the world with approximately 45,000 participants annually. Based on sex and age, individuals walk 30, 40 or 50 km per day for 4 consecutive days. Blood samples were drawn before and after (within 10 min after exercise cessation) the 4 day walking event.

### Experimental design

Blood sampling logistics were performed as described previously [23]. In brief, all participants reported twice to our laboratory, which was located at the start/finish area of the event. Baseline measurements were performed 12–36 h preceding the start (Table 1). Thereafter, the participants walked 30 km a day, for four consecutive days at a self selected pace. Exercise was performed under temperate ambient conditions with daily maximum wet bulb globe temperatures ranging between 24–27 °C. The recovery phase (fluid intake, food intake, sleep) between walking stages was uncontrolled and not monitored. Walking duration was recorded every day, while speed was calculated accordingly. Immediately (within 10 min) after finishing on the fourth day, all baseline measurements were repeated. Heart rate, as part of exercise intensity, was measured during day 1 as described previously [24]. Mean heart rate during exercise was presented in absolute values (beats per minute, (bpm)) and as a percentage of the predicted maximal heart rate. Predicted maximal heart rate (HRmax) was calculated using the Tanaka’s formula: HRmax = 208 - 0.7*age [25]. During the experiment, dry bulb, wet bulb and globe temperatures were measured every 30 min using a portable climate monitoring device (Davis instruments Inc., Hayward, USA), which was positioned at the start/finish area. The wet bulb globe temperature index (WBGT) was calculated using the formula: WBGT = 0.1 (Tdry bulb) + 0.7 (Twet bulb) + 0.2 (Tglobe).

**Table 1:** Demographics, health status and exercise characteristics of volunteers

	Men (*n* = 11)	Women (*n* = 9)
Demographic characteristics		
Age (yr)	81.0 ± 1.2	81.6 ± 2.7
Height (cm)	174 ± 5.2	159 ± 6.7
Weight (kg)	75.8 ± 6.6	55.5 ± 6.6
Body-mass index (kg/m^2^)	25.0 ± 1.6	21.8 ± 1.9
Lean body mass (kg)	57.2 ± 4.7	37.8 ± 5.2
Health status		
Physical activity (hrs/week)	8.5 ± 8.0	5.8 ± 5.9
≥ 5 times/week ≥30 min exercise (%)	73	56
Blood pressure		
Systolic (mmHg)	140 ± 18	146 ± 17
Diastolic (mmHg)	81 ± 11	82 ± 10
Resting heart rate (bpm)	64 ± 20	63 ± 14
Gait speed (km/h)	4.8 ± 0.7	4.5 ± 0.7
Grip strength (kg)	41 ± 8.0	25 ± 4.5
CMV seropositive	6 (55%)	7 (78%)
Use of prescribed medicine		
Anti-hypertensive drugs	2 (18%)	3 (33%)
Statins	2 (18%)	1 (11%)
Analgesics	3 (27%)	0 (0%)
Anti-diabetics	0 (0%)	0 (0%)
Other^a^	2 (18%)	1 (11%)
Pathology		
Hypertension	2 (18%)	3 (33%)
Cardiovascular disease	2 (18%)	0 (0%)
Hypercholestorolemia^b^	2 (18%)	1 (11%)
Diabetes	0 (0%)	0 (0%)
Cancer (not further differentiated)	2 (18%)	1 (11%)
Other^a^	1 (9%)	2 (22%)
Exercise characteristics		
Exercise duration per day (hh:mm)	7:28 ± 1:10	8:07 ± 0:52
Speed (km/h)	4.2 ± 0.1	3.7 ± 0.1
Average heart rate day 1 (bpm)	99.5 ± 11.9	113.6 ± 14.3
Peak heart rate day 1 (bpm)	112.5 ± 13.1	125.0 ± 14.1
Exercise intensity day 1 (% of age-adjusted max. heart rate)	65.7 ± 7.7	75.4 ± 9.6
Fluid balance		
Fluid intake (L)^c^	1.88 ± 0.8	1.98 ± 0.8
Change in body mass (absolute kg)^c^	-0.80 ± 0.9	-0.19 ± 0.6
Change in body mass (relative %)^c^	-1.04 ± 1.1	-0.38 ± 1.0
Change in plasma volume (relative %)^d^	+3.1 ± 3.2	+4.5 ± 3.5

^a^Volunteers who were diagnosed and treated for cancer, rheumatoid arthritis, allergy and glaucoma. ^b^Hypercholesterolemia is defined as total cholesterol levels of >6.5 mmol, as previously diagnosed by a physician. ^c^Daily fluid intake and changes in body mass during walking. ^d^Changes in plasma volume during walking estimated according to Dill and Costill [27]. Data are presented as mean ± standard deviation

### Subject characteristics

Body mass (Seca 888 scale, Hamburg, Germany) and body height were measured and body mass index (BMI) was calculated. A four-point skin fold thickness measurement (biceps, triceps, sub-scapular, supra-iliac) was obtained in order to calculate the lean body mass [26]. Resting heart rate and blood pressure were measured twice using an automated sphygmomanometer (M5-1 intellisense, Omron Healthcare, Hoofddorp, the Netherlands) after 5 min supine rest. Finally, all subjects completed a questionnaire about their physical activity and health status.

### Blood analyses

White blood cell differential counts, hemoglobin and hematocrit levels were directly analyzed on a Sysmex XE-5000 system (Sysmex Corporation, Kobe, Japan). Relative changes in plasma volume were calculated from blood hematocrit and hemoglobin concentrations using Dill and Costill’s equation [27]. C-reactive protein (CRP) and creatinine measurements were measured in batches of frozen sera. Sera aliquots were stored at -20 °C directly after collection and thawed directly before analysis. All analyses were coded and anonymized. Serum CRP and creatinine were both measured on the c16000 Architect (Abbott Diagnostics, Abbott Park, IL).

### Detection of CMV-specific IgG

Serum levels of CMV-specific IgG was essentially done as previously described [7]. In brief, 96-well ELISA plates (Greiner) were coated with lysates of CMV-infected fibroblasts overnight. Lysates of non-infected fibroblasts were used as negative controls. Following coating, dilutions of serum samples were incubated for 1 h. Goat-anti-human IgG was added and incubated for 1 h. Samples were incubated with phosphatase for 15 min, and the reaction was stopped with NaOH. The plates were scanned on a Versamax reader (Molecular Devices). A pool of sera from 3 seropositive individuals with known titers of CMV-specific IgG was used to quantify CMV IgG titers in the test samples.

### Flowcytometry

Peripheral blood mononuclear cells (PBMC) were isolated by density gradient centrifugation with Lymphoprep (Axis-Shield) and stored in -180 °C until staining. PBMCs (10^6^ cells) were stained simultaneously employing 4 different staining panels to asses markers of T cell differentiation, Treg and proliferation, NK cell inhibitory receptors and NK cell activating receptors (Table 2). All cell subsets are expressed as cell counts per liter unless indicated otherwise. Cell counts of these subsets were based on data from the full leucocyte differential in combination with the flowcytometry data. As an example, we used the absolute lymphocyte count from the full leucocyte differential and the percentage of CD3 T cells in the lymphocyte gate as assessed by flowcytometry for calculation of the T cell count. To perform intracellular staining with monoclonal antibodies to Cytotoxic T-lymphocyte-associated protein-4 (CTLA-4), forkhead box P3 (FoxP3) and the proliferation marker ki-67, the cells were first fixed and permeabilized with a FoxP3 staining buffer set (eBioscience). Samples were measured on a LSR-II (BD) and data were analyzed with Kaluza software (Beckman Coulter).

**Table 2:** Overview of staining panels and reagents for flowcytometry

Panel	Mab reagent	Clone	Provider
T cell differentiation	CD3-Efluor 605	okt-03	Ebioscience, San Diego, CA, USA
	CD8-APC-H7	RPA-T8	Ebioscience, San Diego, CA, USA
	CD45RO-FITC	UCHL-1	BD bioscience, San Jose, CA, USA
	CCR7-PE-Cy7	3D12	BD bioscience, San Jose, CA, USA
	CD31-AF647	WM-59	BD bioscience, San Jose, CA, USA
	CD28-AF700	28.2	Biolegend, San Diego, CA, USA
	PD1-PE	EH12.2H7	Biolegend, San Diego, CA, USA
	CD4-PcP	SK3	BD bioscience, San Jose, CA, USA
	CTLA-4-BV421	BNI3	BD bioscience, San Jose, CA, USA
Treg/Proliferation	CD8-PE-Cy7	RPA-T8	BD bioscience, San Jose, CA, USA
	CD25-APC	BC96	Ebioscience, San Diego, CA, USA
	CD45RA-Efluor605	HI100	Ebioscience, San Diego, CA, USA
	CD19-FITC	HD37	Dako, Santa Clara, CA, USA
	CD4-APC-H7	RPA-T4	BD bioscience, San Jose, CA, USA
	FOXP3-PE	PCH101	BD bioscience, San Jose, CA, USA
	Ki-67-PcP-Cy5.5	B56	BD bioscience, San Jose, CA, USA
NK cell	CD16-FITC	3G8	Beckman Coulter, Brea, CA, USA
Inhibitory receptors	CD56-ECD	N901	Beckman Coulter, Brea, CA, USA
	CD3-APC-AF750	UCHT1	Beckman Coulter, Brea, CA, USA
	CD45-KO	J.33	Beckman Coulter, Brea, CA, USA
	CD159c (NKG2C)-PE	134591	R&D Systems, Minneapolis, MN, USA
	CD158b (KIR2DL2/3)-PE-Cy7	GL183	Beckman Coulter, Brea, CA, USA
	CD158e1 (KIR3DL1)-APC	Z27.3.7	Beckman Coulter, Brea, CA, USA
	CD158a (KIR2DL1)-APC-AF700	EB6B	Beckman Coulter, Brea, CA, USA
	CD159a (NKG2A)-PB	Z199	Beckman Coulter, Brea, CA, USA
NK cell	CD16-FITC	3G8	Beckman Coulter, Brea, CA, USA
Activating receptors	CD56-ECD	N901	Beckman Coulter, Brea, CA, USA
	CD3-APC-AF750	UCHT1	Beckman Coulter, Brea, CA, USA
	CD45-KO	J.33	Beckman Coulter, Brea, CA, USA
	CD336 (NKp44)-PE	Z231	Beckman Coulter, Brea, CA, USA
	CD337 (NKp30)-PE-Cy5.5	Z25	Beckman Coulter, Brea, CA, USA
	CD335 (NKp46)-PE-Cy7	BAB281	Beckman Coulter, Brea, CA, USA
	CD314 (NKG2D)-APC	ON72	Beckman Coulter, Brea, CA, USA
	CD244 (2B4) -APC-AF700	C1.7.1	Beckman Coulter, Brea, CA, USA
	CD161-PB	191B8	Beckman Coulter, Brea, CA, USA

### Lipopolysacharide (LPS)-stimulated PBMC cytokine production

PBMCs at 1 x 10^6^ cells/mL were stimulated with 1 μg/mL LPS (Sigma-Aldrich, St. Louis, MO, USA) or left unstimulated. Cells were cultured in polypropylene tubes (BD bioscience) in RPMI with 10% FCS for 24 h. After 24 h, supernatants were collected and stored at -20 °C. Culture supernatants were analyzed for production of the cytokines IL-1β, IL-6, IL-8 and TNF-α by enzyme-linked immunosorbent assay (ELISA, Duoset, R&D system, Minneapolis, MN, USA) according to the manufacturer’s instructions, and read with a Versamax reader (Molecular Devices, Sunnyvale, CA, USA). The assay sensitivity was 8 pg/mL for IL-1β, 31 pg/mL for IL-6 and TNF-α, and 312 pg/mL for IL-8. The net cytokine production was calculated as cytokine production of the stimulated sample minus the cytokine production of the non-stimulated sample.

### Statistical analyses

Statistical analysis of data was carried out using IBM SPSS Statistics 20 (IBM, Chicago, IL, USA) and Graphpad Prism 5 (Graph Pad Software, San Diego, CA, USA). Wilcoxon Signed Rank test, unless indicated otherwise, was used to compare the same volunteers before and after walking. Two-tailed *p*-values of less than 0.05 were considered statistically significant.

## Results

### Subject characteristics

All study participants (*n* = 20) successfully completed the Four Days Marches at a self selected pace (4.0 ± 0.7 km/h). On average the participants walked 7 h and 47 min daily and had an average heart rate during the first day of 106 ± 15 bpm, representing an average exercise intensity of 70 ± 10%. Thus, the walking exercise in these elderly is qualified as a daily bout of moderate intensity exercise for 4 consecutive days. An overview of all subject characteristics is presented in Table 1. An exercise-induced plasma volume expansion of 3.7 ± 3.3% was observed over the 4 days, which coincided with a small, but significant decrease of hematocrit from 0.40 L/L at baseline to 0.38 L/L directly after 4 days of exercise (*p* < 0.0001).

### Effects of acute exercise on the peripheral blood cellular composition

When examining the composition of the peripheral blood compartment, our data show that the walking exercise resulted in a clear leucocytosis with numerical increases of granulocytes, monocytes and lymphocytes (Table 3). When analyzing the lymphocyte compartment, clear numerical increases were noted for T cells and to a lesser extent B cells. In contrast, a decline in the number of NK cells was detected. The numerical increase in T cells was largely due to an increase in CD4+ T cells (Table 3). Although CD8+ T cell numbers showed a statistical significant increase after exercise, the absolute increase was very limited. The mean CD4/CD8 ratio, an age-appropriate value of 3 [9], was not significantly increased by the walking exercise (data not shown).

**Table 3:** General and Immune parameters before and after walking

	Pre-Walking	Post-Walking	*P*-value
Hemoglobin (mmol/L)	8.7 (7.7–9.4)	8.2 (7.3–9.1)	0.0009
Thrombocytes (10^9^/L)	233 (157–318)	234 (160–349)	ns
CRP (mg/L)	1 (1–9)	1 (1–47)	0.0078
Creatinin (μmol/L)	86 (47–147)	100 (54–207)	0.0008
ASAT (U/L)	28 (14–39)	(-)	(-)
ALAT (U/L)	28 (21–46)	(-)	(-)
Leukocytes (10^9^/L)	6.6 (4.6–11.0)	7.7 (5.7–14.3)	0.0002
Neutrophils (10^9^/L)	4.1 (2.4–8.3)	5.1 (2.9–10.4)	0.0008
Eosinophils (10^9^/L)	0.15 (0.03–0.68)	0.18 (0.06–0.78)	0.0166
Basophils (10^9^/L)	0.04 (0.01–0.07)	0.03 (0.02–0.05)	ns
Monocytes (10^9^/L)	0.49 (0.29–0.90)	0.67 (0.40–1.21)	0.0005
Lymphocytes (10^9^/L)	1.57 (1.00–2.21)	1.82 (1.07–2.97)	0.0045
CD3+ T cells (10^9^/L)	0.75 (0.20–1.36)	1.11 (0.48–1.98)	0.0005
CD4+ T cells (10^9^/L)	0.46 (0.06–0.99)	0.59 (0.37–1.62)	0.0007
CD8+ T cells (10^9^/L)	0.15 (0.03–0.45)	0.15 (0.05–0.71)	0.0061
CD19+ B cells (10^9^/L)	0.23 (0.05–0.40)	0.30 (0.09–0.51)	0.0023
CD16 + CD56+ NK cells (10^9^/L)	0.40 (0.21–0.87)	0.31 (0.13–0.70)	0.0178

Medians + range are indicated (*n* = 20). The minimal increase in exercise-induced plasma volume (3.7%) did not influence any of the significant differences found

As carriage of CMV has pronounced effects on the immune system, we compared the effects of the walking exercise between CMV seropositive (*n* = 13) and CMV seronegative subjects (*n* = 7). Although an exercise induced leucocytosis was seen in both CMV seropositive and seronegative individuals, increases in granulocytes, monocytes and lymphocytes were statistically significant in CMV seropositive subjects but did not reach significance in CMV seronegative subjects (Table 4). Lymphocytosis in CMV seropositive subjects was largely caused by T cells, rather CD4 than CD8, and to a lesser extent B cells, whereas NK cell numbers remained unchanged. B cell numbers increased irrespective of CMV serostatus. Interestingly, a decrease of NK cells was seen only in CMV seronegative subjects. Thus, acute exercise induced responses were more clear in CMV seropositive octogenarians.

**Table 4:** Immune parameters before and after walking in CMV seropositive and seronegative participants

	CMV+		CMV-	
	Before	After	Before	After
Hemoglobin^a^ (mmol/L)	8.4 (7.7–9.4)	7.9 (7.3–8.7)**	8.9 (7.9–9.0)	8.7 (7.5–9.1)
CRP (mg/L)	1 (1–9)	1 (1–10)	1 (1–6)	9 (1–47)
Leukocytes (10^9^/L)	6.6 (4.6–11.0)	7.9 (5.7–14.3)**	6.6 (4.8–9.1)	7.2 (6.3–12.3)*
Neutrophils (10^9^/L)	3.8 (2.4–8.3)	5.0 (2.9–10.4)*	4.2 (3.2–6.6)	5.1 (4.2–9.2)*
Eosinophils (10^9^/L)	0.12 (0.03–0.68)	0.19 (0.06–0.78)**	0.17 (0.08–0.28)	0.15 (0.08–0.33)
Basophils (10^9^/L)	0.03 (0.01–0.06)	0.03 (0.02–0.05)	0.05 (0.02–0.07)	0.04 (0.03–0.05)
Monocytes (10^9^/L)	0.48 (0.29–0.69)	0.68 (0.40–1.05)**	0.57 (0.32–0.90)	0.66 (0.42–1.21)
Lymphocytes (10^9^/L)	1.65 (1.45–2.21)	2.19 (1.33–2.97)**	1.37 (1.00–1.92)	1.40 (1.07–2.08)
CD3+ T cells (10^9^/L)	0.77 (0.49–1.36)	1.19 (0.73–1.98)**	0.63 (0.20–1.00)	0.76 (0.48–1.45)
CD4+ T cells (10^9^/L)	0.52 (0.17–0.99)	0.73 (0.40–1.62)**	0.40 (–0.06–0.69)	0.57 (0.37–0.87)
CD8+ T cells (10^9^/L)	0.21 (0.04–0.45)	0.21 (0.06–0.72)*	0.08 (0.04–0.17)	0.10 (0.05–0.19)
CD19+ B cells (10^9^/L)	0.26 (0.09–0.40)	0.35 (0.11–0.51)*	0.10 (0.05–0.40)	0.16 (0.09–0.45)*
CD16 + CD56+ NK cells (10^9^/L)	0.32 (0.21–0.87)	0.31 (0.13–0.70)	0.43 (0.38–0.52)	0.29 (0.22–0.43)*

CMV+ subjects (*n* = 13) and CMV- subjects (*n* = 7). Medians and range are shown. Paired analysis was peformed separately for CMV+ and CMV- subjects before and after excersise. Statistical significance by Wilcoxon signed rank test is indicated as *p < 0.05 or **p < 0.01

^a^Changes in plasma volumes estimated according to Dill and Costill were not significantly different between CMV+ and CMV- subjects

### Effect of acute exercise on T cells

**Fig. 1:**
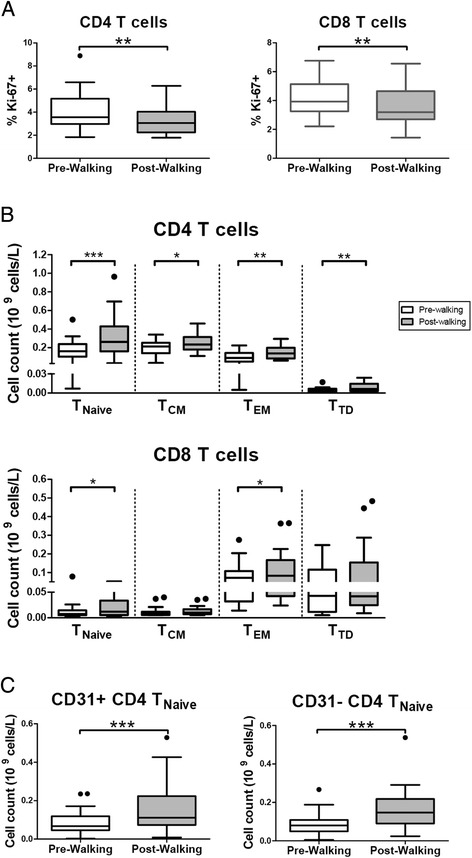
Exercise reduces rates of T cell proliferation and leads to redistribution of T cell subsets. **a** Rates of CD4 (left panel) and CD8 (*right panel*) proliferation before (Pre-Walking) and after exercise (Post-Walking) assessed by Ki-67 expression using flow-cytometry. Percentages of Ki-67 expressing cells within the CD4 and CD8 populations are shown. **b** Enumeration of CD4 (upper panel) and CD8 (lower panel) T cell differentiation subsets based on CD45RO and CCR7 expression Pre- and Post-Walking. Mean (+/- SEM) numbers (10^9^ cells/L) of naïve T cells (T_Naïve_), central memory T cells (T_CM_), effector memory T cells (T_EM_) and terminally differentiated T cells (T_TD_) are shown. **c** Mean (+/- SEM) numbers (10^9^ cells/L) of recent thymic emigrants defined as CD31 + CD4 + T_Naïve_ and the central naïve CD31-CD4+ T_Naïve_ subsets. Statistical significance by Wilcoxon signed rank test is indicated as *p < 0.05, **p < 0.01, ***p < 0.001

We next examined the proliferative properties of circulating immune cells as measured by Ki-67 expression. Post walking, both CD4+ and CD8+ T cells demonstrated reduced percentages of proliferating cells (Fig. 1a). Similar observations were seen in CMV seropositive and seronegative subjects (Additional file 1: Figure S1a). Thus, the data suggest an exercise-induced increase of T cells with low proliferative capacity [20].

To determine if certain T cell populations respond differently, we next investigated exercise-induced changes in CD4+ and CD8+ differentiation subsets. A flow-cytometric analysis employing CD45RO and CCR7 was applied to identify naïve T cells (T_Naïve_), central memory T cells (T_CM_), effector memory T cells (T_EM_) and terminally differentiated T cells (T_TD_) [28]. Numerical changes of differentiation subsets were most profound in the CD4+ compartment (Fig. 1b) and appeared to be linked to CMV carriage (Additional file 1: Figure S1b). Although significant increases were noted for all four differentiation subsets, the most profound increase was noted in the CD4+ T_Naïve_ subset (Fig 1b). Interestingly, we also noted a significant increase of CD4+ T_Naïve_ in CMV seronegative subjects (Additional file 1: Figure S1b). When analysed for the contribution of recent thymic emigrants (RTE), defined as CD31^pos^ CD4 + T_Naïve_ and the central naïve CD31^neg^ CD4 + T_Naïve_ subsets, we found both subsets significantly increased [29] (Fig. 1c). In CMV seronegative subjects the rise in CD4+ T_Naïve_ appeared due to the CD31^neg^ CD4 + T_Naïve_ subset (Additional file 1: Figure S1c)_._ Moreover, the naïve CD4 + CD25^dim^ subset, recently described to develop in secondary lymphoid organs upon TCR priming, was found significantly increased ([30], data not shown). The combined data suggest the exercise-induced response of CD4 + T_Naïve_ and CD4+ memory subsets (T_EM_ >T_TD_ > T_CM_).

In the CD8+ compartment, slight but significant numerical increases were noted for both the CD8 T_EM_ and the CD8 + T_Naïve_ subsets, whereas the T_CM_ and the T_TD_ subsets were not changed (Fig. 1b). These changes appeared associated with CMV carriage (Additional file 1: Figure S1b). Taken together, our findings show an increase of peripheral naive CD4+ T cells particularly in response to exercise. Carriage of CMV is largely associated with enhanced numbers of both CD4 and CD8 subsets.

### No effect of acute exercise on markers of T cell exhaustion

As we noted numerical increases in particularly the CD4+ T_EM_ and the T_TD_ subsets and to a lesser extent the CD8 T_EM_, but not the CD8 T_TD_ subset, we examined these subsets for expression of the exhaustion markers CTLA-4 and PD-1 [8]. Frequencies of CTLA-4 and PD-1 in the CD4+ T_EM_ and the T_TD_ subsets were largely comparable before and after the walking exercise; although a slight decrease of PD-1 expressing cells in the T_EM_ subset was detected (Additional file 2: Figure S2). In the CD8+ T_EM_ and the T_TD_ subsets, where only modest or no numerical increases were noted, respectively, only slight increases in the frequencies of CTLA4-, but not PD1-expressing cells were observed. Expression of these markers was generally lacking on T_Naïve_ and T_CM_ (data not shown). Thus, the walking exercise does not affect markers of immune exhaustion of either CD4 or CD8 T_EM_ and T_TD_ subsets.

### Acute exercise-induced changes in nTreg but not memory Treg subsets

**Fig. 2:**
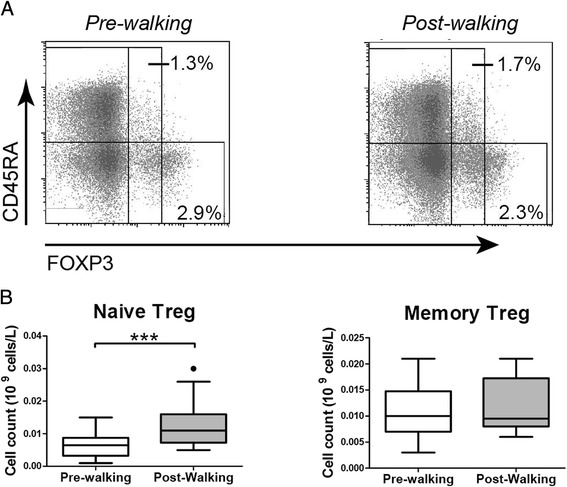
Exercise-induced changes of naïve Treg, but not memory Treg. **a** Representative CD45RA and FoxP3 staining in CD4 T cells before and after exercise (Pre-walking and Post-walking). Naïve Treg cells are identified by CD45RA + Foxp3^dim^ and memory Treg cells by CD45RA-FoxP3^high^ [31]. **b** Mean (+/- SEM) numbers (10^9^ cells/L) of Naïve Treg and Memory Treg Pre-walking and Post-walking. Statistical significance by Wilcoxon signed rank test is indicated as ***p < 0.001

Based on CD45RA and FoxP3 expression [31], CD45RA + FoxP3^low^ naïve (resting) Treg cells (nTreg) and CD45RA-FoxP3^high^- memory (activated) Treg cells (memTreg) were identified in the peripheral blood of elderly walkers (Fig. 2a). Post exercise, a clear numerical increase of nTreg was observed whereas the numbers of memTreg remained stable (Fig. 2b). The increase in nTreg was seen irrespective of CMV serostatus, although the increase was more clear in CMV seropositive subjects (Additional File 3, Figure S3). Thus, as seen with the conventional CD4+ T_Naïve_ cells, the peripheral numbers of nTregs in elderly walkers also increased in response to acute exercise. In contrast, whereas exercise led to increases in conventional CD4+ memory T cells (Fig. 1b), exercise did not induce numerical increases of memTreg (Fig 2b).

### Effects of acute exercise on NK cells

**Fig. 3:**
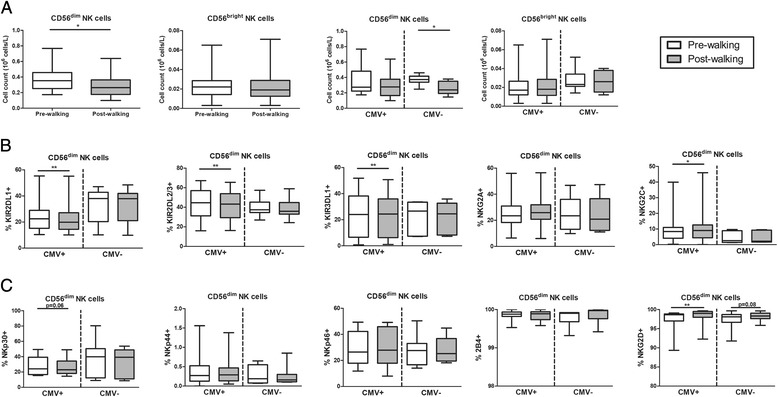
Exercise-induced changes in NK cells. **a** Mean (+/- SEM) numbers (10^9^ cells/L) of CD56^dim^ and CD56^bright^ NK subsets Pre-walking and Post-walking in the study population (*n* = 20, left panels) and when stratified for CMV serostatus (CMV+; *n* = 13, CMV-; *n* = 7, right panels). **b** Percentages of KIR2DL1+, KIR2DL2/3+, KIR3DL1+, NKG2A+ and NKG2C+ NK cells within CD56^dim^ NK cells Pre-walking and Post-walking in CMV+ and CMV- subjects. **c** Percentages of NKp30+, NKp44, NKp46+,2B4+, NKG2D+ within CD56^dim^ NK cells Pre-walking and Post-walking in CMV+ and CMV- subjects. Statistical significance by Wilcoxon signed rank test is indicated as *p < 0.05, **p < 0.01

Peripheral NK cell numbers were found reduced after exercise which was caused by a numerical decline of CD56^dim^ but not CD56^bright^ NK cells [15] (Table 3 and Fig. 3a). Notably, the decrease in CD56^dim^ NK cells was seen in CMV seronegative donors only (Fig 3a). As CD56^dim^ NK cells are most frequent in the blood (90% of total NK cells), we next investigated exercise-induced changes in the expression of inhibitory and activating NK receptors by this subset. In CMV seronegative subjects, we found comparable frequencies of cells positive for inhibitory receptors (KIR2DL1, KIR2DL2/3, KIR3DL1, NKG2A and NKG2C) and activating receptors (NKp30, NKp44, NKp46, 2B4 and NKG2D) within CD56^dim^ NK cells before and after walking (Fig. 3bc). However, in CMV seropositive subjects, exercise did modulate frequencies of NK cells with inhibitory and activating receptors. More specifically, KIR2DL1-, KIR2DL2/3- and KIR3DL1-positive cells decreased with exercise, but frequencies of NKG2C-positive cells were increased (Fig. 3b). Also, frequencies of activating receptor NKG2D+ cells were found increased (Fig. 3c). Thus, the walking exercise led to a numerical decline of CD56^dim^ NK cells in CMV seronegative subjects. In CMV seropositive subjects the walking exercise did not seem to affect the numbers of NK cells but down-modulated expression of most inhibitory receptors whereas the expression of activating receptors was largely unchanged, suggesting a less inhibited phenotype as a net result.

### Exercise leads to higher LPS-stimulated cytokine production by PBMC

**Fig. 4:**
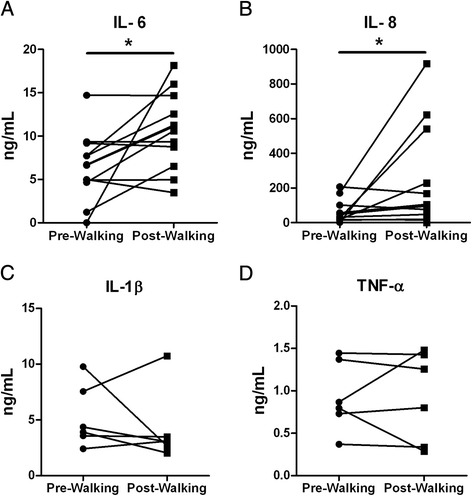
LPS-stimulated PBMC cytokine production before and after walking. Cytokines (**a**) IL-6, (**b**) IL-8, (**c**) IL-1β and (**d**) TNFα in the 24 h supernatant of LPS-stimulated PBMC isolated pre and post walking. Individual pre- and post- samples are connected by lines. Cytokines in supernatants were assessed with ELISA and expressed as ng/mL. The assay sensitivity was 8 pg/mL for IL-1β, 31 pg/mL for IL-6 and TNF-α, and 312 pg/mL for IL-8. The net cytokine production was calculated as cytokine production of the stimulated sample minus the cytokine production of the non-stimulated sample. IL-6 and IL-8 were assessed in 12 walkers (8 males and 4 females). IL-1β and TNF-α were assessed in 6 walkers (4 males and 2 females). Statistical significance by Wilcoxon signed rank test is indicated as *p < 0.05

We next investigated the effects of exercise on PBMC function. Hereto, we analysed the LPS stimulated production of IL-1β, TNFα, IL-6 and IL-8 by PBMC before and after walking. The data show increases in production of IL-6 and IL-8, but not IL-1β and TNFα (Fig. 4). Similar data were obtained in CMV seropositive and seronegative subjects (data not shown).

## Discussion

Our main finding is that acute exercise induced changes in immune cell numbers and functions in an exceptional cohort of octogenarian walkers. A clear response of CD4+ T cells, rather than CD8+ T cells or NK cells to exercise was noted. Moreover, the response was dominated by numerical increases of naïve CD4+ subsets.

Effects of exercise were evaluated in a paired sample design study enumerating a comprehensive set of peripheral cellular traits, before and directly after the walking event. As it is known that most exercise-induced changes in immune cell counts return to pre-exercise levels within a few hours, we emphasise that our data likely reflect the effect of the final day of exercise. We report on clear exercise-induced numerical increases of granulocytes, monocytes and lymphocytes. A retrospective analysis showed that these changes were associated with CMV carriage, thereby confirming the notion that infection history not only impacts the composition of the peripheral blood compartment but also the response to exercise, as recently suggested [21, 22].

Previous studies in adult subjects show that CD8 effector memory T cells and NK cells are the most exercise responsive lymphocytes [19, 20]. Preferential mobilization of these cells from the marginal pool is caused by increases in haemodynamic shear forces and by the relatively high expression of β-adrenergic receptors on these cells, leading to detachment of lymphocytes from endothelial cells upon catecholamine stimulation [22]. Following exercise cessation, both NK cells and CD8+ memory T cells quickly reallocate to the tissues. Only recently, it was documented that CMV latency enhances the exercise-induced mobilization of CD8 effector memory T cells in adult and middle aged (50–64 years old) subjects [21, 22, 32]. Interestingly, mobilization of NK cells was less pronounced in both adult and middle aged CMV carriers, suggesting that CMV infection may impair their mobilization [22, 33]. Moreover, CMV carriage delayed the egress of both CD8 T_EM_ cells and NK cells to the tissues. The latter may be due to impaired β-adrenergic receptor signalling in CMV carriers [34].

Our study revealed a very modest increase of CD8 T_EM_ cells in CMV carriers and a decrease of CD56^dim^ NK cells in CMV non-carriers. This may be explained by the timing of the blood sampling, which was done within 10 min after exercise cessation, in the early recovery phase. The selective decrease of CD56^dim^ NK cells may indeed suggest a rapid distribution of these cells to the tissues. In contrast, we found proportions of NKG2C+ CD56^dim^ NK cells increased after exercise in CMV seropositive subjects, which would be in line with their delayed egress due to catecholamine insensitivity [22, 34]. Thus, also in octogenarians, CMV carriage may delay the egress of CD8 T_EM_ cells and ‘memory-like’ NK cells to the tissues.

Latent CMV infection may also increase the mobilization of CD4+ effector memory T cells, albeit to a lesser extent than CD8 T cells [21, 32]. Our octogenarian walkers showed limited increases in CD4+ effector memory and in terminally differentiated effector memory T cells in response to exercise. This may be explained by the notion that most memory T cells are retained in peripheral tissues as tissue resident memory T cells [35]. In line with this, markers of immune exhaustion, such as PD-1 and CTLA-4, on memory subsets were unchanged.

Previously, the exercise induced mobilization of CD8+ naïve/early differentiated cells was found to be impaired in middle aged adults, irrespective of CMV serostatus [32]. Data on naïve CD4+ T cell mobilization in middle aged and elderly individuals are scarce. We here report on a robust response of naïve CD4+ T cells in our octogenarian cohort of habitual walkers. Numerical increases of naïve CD4+ T cells were seen irrespective of CMV status. In contrast, the response of naïve CD8 T cells was very limited and is in line with the higher turn-over rate of naïve CD8 T cells as a consequence of ageing and or frequent environmental challenges [7, 36–39]. Conversely, human CD4 naïve T cells are better maintained with age due to peripheral homeostatic proliferation mechanisms involving IL-2, a phenomenon not seen with naïve CD8 T cells [30, 40, 41]. The exercise-induced increases of CD4+ naïve T cells suggests that these cells are retained in the marginal pool and/or lymphoid tissues on to high age.

Few studies have investigated effects of exercise on diverse Treg subsets in elderly cohorts. We here report on exercise-induced changes of naïve/resting regulatory T cells but not activated/memory Treg. The increase in naïve Treg suggests, similar to the RTE and central naïve CD4+ T cells, the maintenance of these subsets on to high age in niches of the primary and secondary lymphoid organs. In contrast, memory Treg, even more so than conventional memory T cells, appear to be retained in the tissues [42].

We found serum CRP levels elevated in response to exercise. This is likely the result of higher levels of systemic IL-6. Indeed, a higher IL-6 and IL-8 production capacity upon LPS-stimulation of PBMC was seen after walking. Yet, production levels of IL-1β and TNFα were unaltered. The higher production levels of IL-6 and IL-8 may reflect the higher number of monocytes in the PBMC fraction after exercise but do not explain why the production capacity of IL-1β and TNFα were not elevated. An age-associated reduced inflammasome activation may underlie the reduced IL-1β and TNFα production capacity of elderly monocytes; a notion that would merit further investigation.

Our study cohort was obviously biased for physical fitness, as the participants were able to complete 4 consecutive days of moderate intensity exercise. The participants, however, were not selected as healthy individuals and 14 out of the 20 participants had at least one disease for which they were treated. Our cohort thus represents a selection of habitual elderly walkers who exercise in spite of having a disease. Yet, baseline values of both innate and adaptive immune cells compare well to values obtained in a healthy elderly cohort who were selected for their health ([9], data not shown).

Importantly, although our study was not designed and powered to definitely conclude on the effects of CMV carriage on the response to exercise, a retrospective analysis already revealed clear effects of CMV carriage on amplitude and/or kinetics of peripheral immune markers in this small cohort of octogenarians. Clearly, further studies in independent elderly cohorts are required to assess the effects of CMV carriage on acute exercise induced immune cell changes.

## Conclusion

Acute exercise induced changes in immune cell numbers and functions in a group of octogenarian walkers. The massive exercise-induced response of naïve CD4+ T cells was most remarkable and adds to the notion of naïve CD4+ maintenance on to high age. The functional consequences of these changes for mobilization of immune responses to novel (and previously encountered) antigens remain to be established.

## Additional files

Additional file 1: Figure S1. Exercise-induced T cell proliferation rates and T cell subset redistribution in CMV+ and CMV- subjects. (a) Rates of CD4 (left panel) and CD8 (right panel) proliferation before (Pre-Walking) and after exercise (Post-Walking) assessed by Ki-67 expression using flow-cytometry. Percentages of Ki-67 expressing cells within the CD4 and CD8 populations are shown. (b) Enumeration of CD4 and CD8 T cell differentiation subsets based on CD45RO and CCR7 expression Pre- and Post-Walking in CMV seropositive subjects (upper panel, *n* = 13) and CMV seronegative subjects (lower panel, *n* = 7). Mean (+/- SEM) numbers (10^9^ cells/L) of naïve T cells (T_Naïve_), central memory T cells (T_CM_), effector memory T cells (T_EM_) and terminally differentiated T cells (T_TD_) are shown. (c) Mean (+/- SEM) numbers (10^9^ cells/L) of recent thymic emigrants defined as CD31 + CD4 + T_Naïve_ and the central naïve CD31-CD4+ T_Naïve_ subsets in CMV+ and CMV- subjects. Statistical significance by Wilcoxon signed rank test is indicated as **p* < 0.05, ***p* < 0.01. (DOCX 995 kb)
Additional file 2: Figure S2. Exercise does not induce PD-1 and CTLA-4 on CD4 and CD8 memory T cells. (a) Percentages of PD-1 expressing cells in CD4 (left panel) and CD8 (right panel) effector memory T cells (T_EM_) and terminally differentiated T cells (T_TD_) pre- (white) and post-walking (grey). (b) Percentages of CTLA-4-expressing cells in CD4 (left panel) and CD8 (right panel) effector memory T cells (T_EM_) and terminally differentiated T cells (T_TD_) pre- (white) and post-walking (grey). (DOCX 775 kb)
Additional file 3 Figure S3. Exercise-induced changes of naïve Treg, but not memory Treg. (a) Mean (+/- SEM) numbers (10^9^ cells/L) of Naïve Treg and Memory Treg Pre-walking and Post-walking in CMV+ (*n* = 13) and CMV- (*n* = 7) subjects. Statistical significance by Wilcoxon signed rank test is indicated as **p* < 0.05 and ***p* < 0.01. (DOCX 739 kb)

## Abbreviations

BMI: Body mass index
bpm: Beats per minute
CCR7: C-C chemokine receptor type 7
CRP: C-reactive protein
CTLA-4: Cytotoxic T-lymphocyte-associated protein 4
ELISA: Enzyme-linked immunosorbent assay
Foxp3: Forkhead box P3
HRmax: Maximal heart rate
IL-1β: Interleukin-1β
IL-6: Interleukin-6
IL-8: Interleukin-8
LPS: Lipopolysacharide
NK: Natural killer
PBMC: Peripheral blood mononuclear cells
PD-1: Programmed cell death protein 1
RTE: Recent thymic emigrants
T_CM_: Central memory T cells
TCR: T cell receptor
T_EM_: Effector memory T cells
T_Naïve_: Naïve T cells
TNF-α: Tumor necrosis factor-α
Treg: Regulatory T cell
T_TD_: Terminally differentiated T cells
WBGT: Wet bulb globe temperature
