# Supplementary material for: Changes in peripheral immune cell numbers and functions in octogenarian walkers – an acute exercise study
Source: Immun Ageing. 2017 Feb 22;14:5. doi: 10.1186/s12979-017-0087-2 (PMC5322590; doi:10.1186/s12979-017-0087-2)
Supplement: Additional file 1: Figure S1. — Exercise-induced T cell proliferation rates and T cell subset redistribution in CMV+ and CMV- subjects. (a) Rates of CD4 (left panel) and CD8 (right panel) proliferation before (Pre-Walking) and after exercise (Post-Walking) assessed by Ki-67 expression using flow-cytometry. Percentages of Ki-67 expressing cells within the CD4 and CD8 populations are shown. (b) Enumeration of CD4 and CD8 T cell differentiation subsets based on CD45RO and CCR7 expression Pre- and Post-Walking in CMV seropositive subjects (upper panel, n = 13) and CMV seronegative subjects (lower panel, n = 7). Mean (+/- SEM) numbers (109 cells/L) of naïve T cells (TNaïve), central memory T cells (TCM), effector memory T cells (TEM) and terminally differentiated T cells (TTD) are shown. (c) Mean (+/- SEM) numbers (109 cells/L) of recent thymic emigrants defined as CD31 + CD4 + TNaïve and the central naïve CD31-CD4+ TNaïve subsets in CMV+ and CMV- subjects. Statistical significance by Wilcoxon signed rank test is indicated as *p < 0.05, **p < 0.01. (DOCX 995 kb) [file 12979_2017_87_MOESM1_ESM.docx]

**Additional file 1: Figure S1**


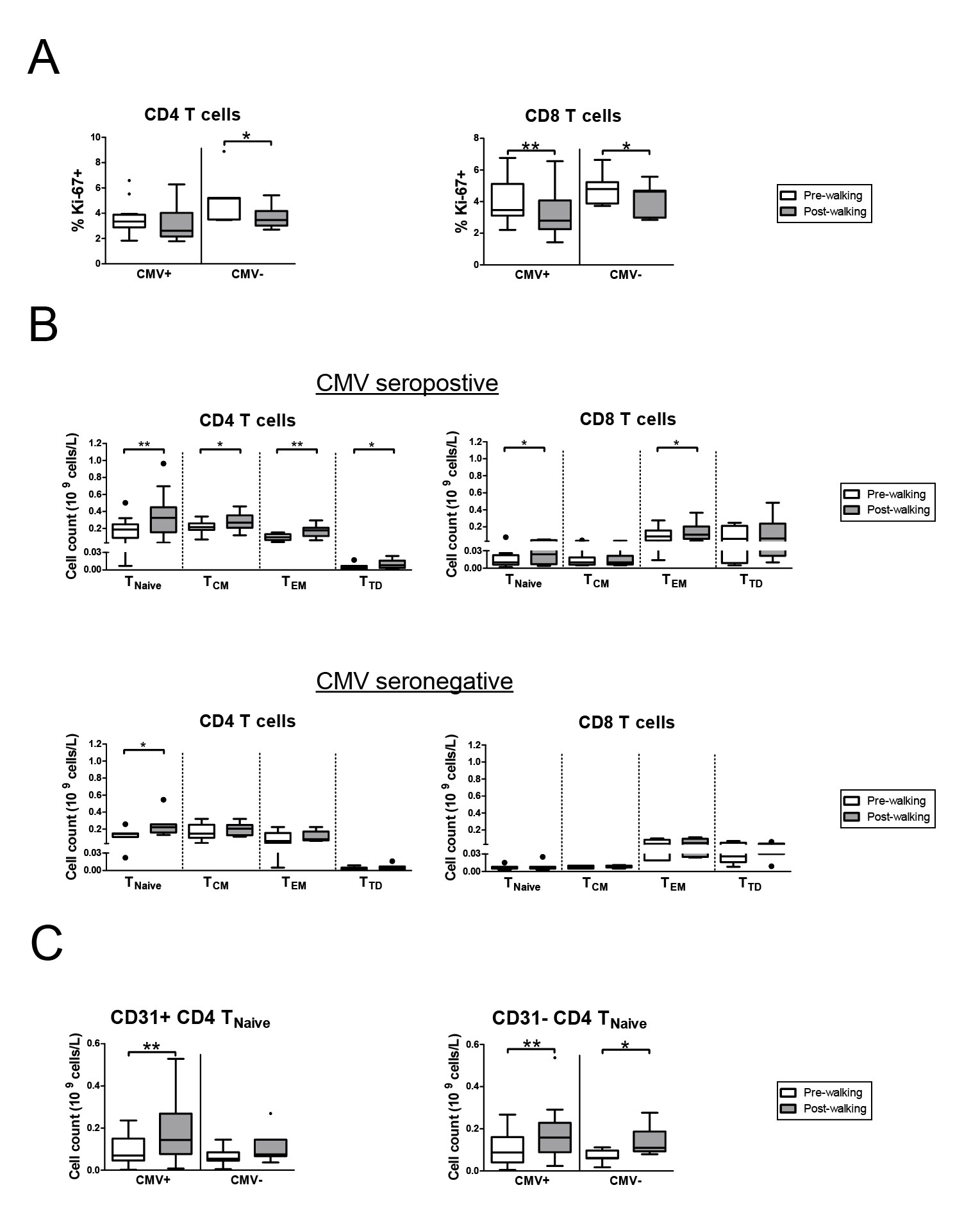


**Figure S1. Exercise-induced T cell proliferation rates and T cell subset redistribution in CMV+ and CMV- subjects.** (a) Rates of CD4 (left panel) and CD8 (right panel) proliferation before (Pre-Walking) and after exercise (Post-Walking) assessed by Ki-67 expression using flow-cytometry. Percentages of Ki-67 expressing cells within the CD4 and CD8 populations are shown. (b) Enumeration of CD4 and CD8 T cell differentiation subsets based on CD45RO and CCR7 expression Pre- and Post-Walking in CMV seropositive subjects (upper panel, n =13) and CMV seronegative subjects (lower panel, n = 7). Mean (+/- SEM) numbers (10^9^ cells/L) of naïve T cells (T_Naïve_), central memory T cells (T_CM_), effector memory T cells (T_EM_) and terminally differentiated T cells (T_TD_) are shown. (c) Mean (+/- SEM) numbers (10^9^ cells/L) of recent thymic emigrants defined as CD31+CD4+T_Naïve_ and the central naïve CD31-CD4+ T_Naïve_ subsets in CMV+ and CMV- subjects. Statistical significance by Wilcoxon signed rank test is indicated as * p<0.05, ** p<0.01.
