# Supplementary material for: Changes in peripheral immune cell numbers and functions in octogenarian walkers – an acute exercise study
Source: Immun Ageing. 2017 Feb 22;14:5. doi: 10.1186/s12979-017-0087-2 (PMC5322590; doi:10.1186/s12979-017-0087-2)
Supplement: Additional file 2: Figure S2. — Exercise does not induce PD-1 and CTLA-4 on CD4 and CD8 memory T cells. (a) Percentages of PD-1 expressing cells in CD4 (left panel) and CD8 (right panel) effector memory T cells (TEM) and terminally differentiated T cells (TTD) pre- (white) and post-walking (grey). (b) Percentages of CTLA-4-expressing cells in CD4 (left panel) and CD8 (right panel) effector memory T cells (TEM) and terminally differentiated T cells (TTD) pre- (white) and post-walking (grey). (DOCX 775 kb) [file 12979_2017_87_MOESM2_ESM.docx]

**Additional file 2: Figure S2**

**
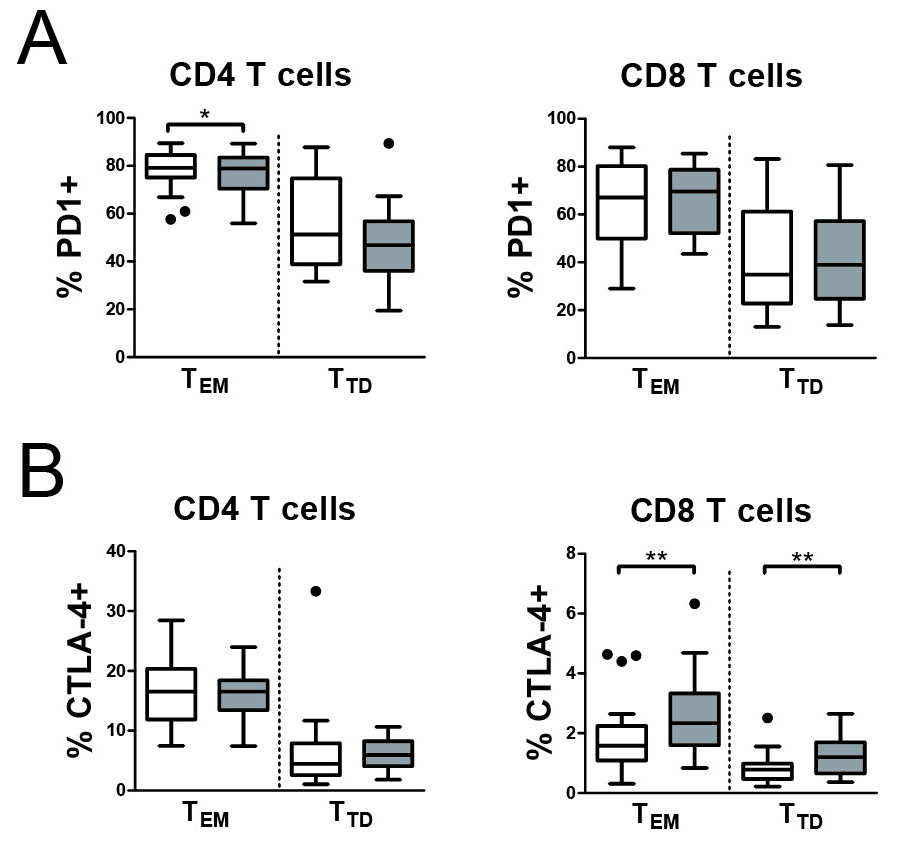
**

**Figure S2. Exercise does not induce PD-1 and CTLA-4 on CD4 and CD8 memory T cells.** (a) Percentages of PD-1 expressing cells in CD4 (left panel) and CD8 (right panel) effector memory T cells (T_EM_) and terminally differentiated T cells (T_TD_) pre- (white) and post-walking (grey). (b) Percentages of CTLA-4-expressing cells in CD4 (left panel) and CD8 (right panel) effector memory T cells (T_EM_) and terminally differentiated T cells (T_TD_) pre- (white) and post-walking (grey).
