# Supplementary material for: Changes in peripheral immune cell numbers and functions in octogenarian walkers – an acute exercise study
Source: Immun Ageing. 2017 Feb 22;14:5. doi: 10.1186/s12979-017-0087-2 (PMC5322590; doi:10.1186/s12979-017-0087-2)
Supplement: Additional file 3 Figure S3. — Exercise-induced changes of naïve Treg, but not memory Treg. (a) Mean (+/- SEM) numbers (109 cells/L) of Naïve Treg and Memory Treg Pre-walking and Post-walking in CMV+ (n = 13) and CMV- (n = 7) subjects. Statistical significance by Wilcoxon signed rank test is indicated as *p < 0.05 and **p < 0.01. (DOCX 739 kb) [file 12979_2017_87_MOESM3_ESM.docx]

**Additional file 3; Figure S3**

**
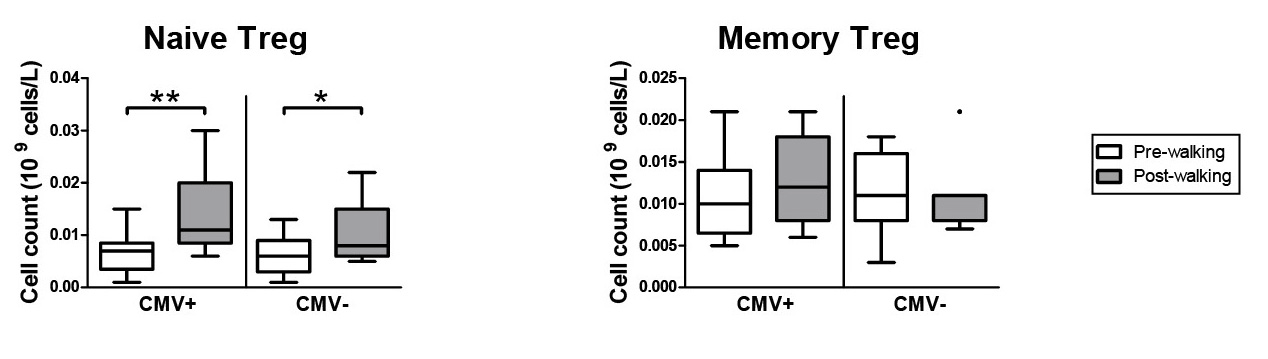
**

**Figure S3.** **Exercise-induced mobilization of naïve Treg, but not memory Treg**. (a) Mean (+/- SEM) numbers (10^9^ cells/L) of Naïve Treg and Memory Treg Pre-walking and Post-walking in CMV+ (n = 13) and CMV- (n = 7) subjects. Statistical significance by Wilcoxon signed rank test is indicated as *p<0.05 and ** p<0.01.
